# Supplementary material for: Identification and validation of intergeneric hybrids between Saccharum officinarum and Erianthus rockii using molecular and cytogenetic tools
Source: PLoS One. 2025 May 29;20(5):e0324128. doi: 10.1371/journal.pone.0324128 (PMC12121808; doi:10.1371/journal.pone.0324128)
Supplement: Attachment — Submitted filename: Response to Reviewers.docx [file pone.0324128.s003.docx]

**Response to Reviewer 1 Comments**

**Major comment:** I have only a one major comment regarding to the study design. The three methods applied in this study have been used in other studies separately as cited (Cai et al. 2005, Aitken et al. 2007, Nair et al. 1999, Devarumath et al. 2012; Lu et al. 2012 and Lin et al. 2013). Although the authors optimized ARMS and HRM for simplified setup and shortened analysis time, I did not see the solid needs to apply all three methods as an integral. To me, the electrophoretogram of figure 5 is already evident for assessing the validity of hybrids. Could the authors explain and elaborate more why the three methods should be applied collectively? Is there any limitation of each method? Also, the GISH technique reveals the composition and the structure of chromosomes and in this case perfectly showed the n+n compositions of true hybrids. However, aneuploid is common in plants which are hybridized or mutated. Did the author considered the aneuploid issues when using this cytogenetic tool?

**Response:** Thank you for your comments. In response to your three queries, I will address them individually.

1. In general, I agree with your observation that the authenticity of hybrids can be verified solely through ARMS-PCR using the developed molecular marker. However, for any newly developed molecular marker, its reliability should be validated through additional methods. Once the reliability of a newly developed molecular marker has been confirmed through multiple methods, it becomes redundant to apply all three methods simultaneously in every case. Only ARMS-PCR, based on the developed molecular markers, is required for hybrid authenticity verification.

2. All three techniques have limitations. ARMS-PCR requires high specificity for primers and amplification conditions, necessitating numerous pre-experiments to identify optimal conditions. HRM equipment and reagents are relatively costly. The GISH experimental process is complex and not suitable for large-scale identification.

3. *S. officinarum* is autopolyploid with 2n = 8x = 80, while *E. rockii* is diploid with 2n = 30. The number of gametic chromosomes produced during meiosis is typically euploidy halved, so the chromosome number of F_1_ hybrids typically conforms to the n + n genetic pattern. However, when F_1_ hybrids are crossed further, they may produce gametes with aneuploidy halving the number of chromosomes, and the chromosome numbers of their offspring will exhibit deviations from the n + n pattern.

**Minor comments 1:** The authority of Saccharum officinarum should be Linnaeus. Is that "Badila" is a cultivar or a line? If so, the cultivar name should be enclosed in a pair of single quotation marks i.e. 'Badila', following the International Code of Nomenclature for Cultivated Plants (ICNCP). Also, please do so for "Yunnan 15-7" if this situation is applied.

**Response:** Agree. “Badila” is a cultivar, and “Yunnan 15-7” is a wild material found in the fields of Yunnan, China. We have, accordingly, enclosed all “Badila” and “Yunnan 15-7” in the manuscript with a pair of single quotation marks. The changes can be found in the revised manuscript lines 111-119, 158, 200-201, 225, 229, 239, 269, 298, 307-317 and 335.

**Minor comments 2:** In line 281, the titles of Fig 6b ("the normalized fluorescence curves") and Fig 6c ("the difference plot melt curve") are not consistent with the one in Figure 6 ("Normalized Melting Curves" and "Normalized and Temp-Shifted Difference Curves"). Please be consistent for easy comprehension.

**Response:** Agree. We have, accordingly, modified the content in the HRM assay identification of true and false F_1_ hybrids of the Results to emphasize this point. Line 281 to 282 in the revised manuscript this change can be found.

**Minor comments 3:** In Table 1, the primer names (R-D2 5'-3' RI-Dian4 5'-3') are not identical with those (FI-Er, RI-So) in the main text. Please check and revise.

**Response:** Agree. We have, accordingly, modified the primer names in Table 1 to emphasize this point. Table 1 in the revised manuscript this change can be found.

**Response to Academic Editor Comments**

**Comment 1:** Please ensure that your manuscript meets PLOS ONE's style requirements, including those for file naming. The PLOS ONE style templates can be found at <https://journals.plos.org/plosone/s/file?id=wjVg/PLOSOne_formatting_sample_main_body.pdf> and <https://journals.plos.org/plosone/s/file?id=ba62/PLOSOne_formatting_sample_title_authors_affiliations.pdf>

**Response:** Agree. We have, accordingly, changed the font size of headings at all levels to emphasize this point. In the revised manuscript this change can be found.

**Comment 2:** We note that the grant information you provided in the ‘Funding Information’ and ‘Financial Disclosure’ sections do not match. When you resubmit, please ensure that you provide the correct grant numbers for the awards you received for your study in the ‘Funding Information’ section.

**Response:** Agree. We have, accordingly, added the following funding statement content in the ‘Funding Information’ section to emphasize this point. The following text is the funding statement: This research was funded by GDAS’ Project of Science and Technology Development (2022GDASZH-2022010102), the GDAS’s Project of Technical Innovation and Incubation Service Platform Construction (2021GDASYL 20210301001). The funders had no role in study design, data collection and analysis, decision to publish, or preparation of the manuscript.

**Comment 3:** Thank you for stating the following in the Acknowledgments Section of your manuscript:
The authors express their sincere gratitude to Professor Zuhu Deng from Fujian Agriculture and Forestry University for his invaluable assistance in conducting the GISH assay. This research was funded by GDAS’ Project of Science and Technology Development (2022GDASZH-2022010102), the GDAS’s Project of Technical Innovation and Incubation Service Platform Construction (2021GDASYL 20210301001) and was supported by Provincial scientific research institutions stability support sub-project in 2020 "Breeding and construction of healthy seedling propagation system of new sugarcane varieties (lines)".

We note that you have provided funding information that is not currently declared in your Funding Statement. However, funding information should not appear in the Acknowledgments section or other areas of your manuscript. We will only publish funding information present in the Funding Statement section of the online submission form.
Please remove any funding-related text from the manuscript and let us know how you would like to update your Funding Statement. Currently, your Funding Statement reads as follows:
The author(s) received no specific funding for this work.

Please include your amended statements within your cover letter; we will change the online submission form on your behalf.

**Response:** Agree. We have, accordingly, removed funding-related text in the Acknowledgments section to emphasize this point. Line 400 to 402 in the revised manuscript this change can be found.

**Comment 4:** We note that your Data Availability Statement is currently as follows: All relevant data are within the manuscript and its Supporting Information files.

Please confirm at this time whether or not your submission contains all raw data required to replicate the results of your study. Authors must share the “minimal data set” for their submission. PLOS defines the minimal data set to consist of the data required to replicate all study findings reported in the article, as well as related metadata and methods (<https://journals.plos.org/plosone/s/data-availability#loc-minimal-data-set-definition>).

For example, authors should submit the following data:

- The values behind the means, standard deviations and other measures reported;
- The values used to build graphs;
- The points extracted from images for analysis.

Authors do not need to submit their entire data set if only a portion of the data was used in the reported study.

If your submission does not contain these data, please either upload them as Supporting Information files or deposit them to a stable, public repository and provide us with the relevant URLs, DOIs, or accession numbers. For a list of recommended repositories, please see <https://journals.plos.org/plosone/s/recommended-repositories.>
**Response:** Agree. We have, accordingly, presented raw data of Table 2 as a supporting information S1 File to emphasize this point. Line 613 to 614 in the revised manuscript this change can be found.
